# The effectiveness of a dynamic seat cushion in preventing neck and low-back pain among high-risk office workers: a 6-month cluster-randomized controlled trial

**DOI:** 10.5271/sjweh.4184

**Published:** 2024-10-01

**Authors:** Sirinant Channak, Erwin M Speklé, Allard J van der Beek, Prawit Janwantanakul

**Affiliations:** 1 Department of Physical Therapy, Faculty of Allied Health Sciences, Chulalongkorn University, Bangkok, Thailand.; 2 Arbo Unie Occupational Health Service, Nieuwegein, The Netherlands.; 3 Department of Public and Occupational Health, Amsterdam Public Health Research Institute, Amsterdam UMC, Vrije Universiteit Amsterdam, Amsterdam, The Netherlands.

**Keywords:** discomfort, dynamic sitting, muscle activity, office worker, postural shift

## Abstract

**Objectives:**

This study evaluated the effectiveness of the promotion of postural shift intervention using a dynamic seat cushion on the 6-month incidence of neck and low-back pain among high-risk office workers.

**Methods:**

In a cluster-randomized controlled trial (RCT), 133 office workers were randomly assigned, at cluster level, to intervention (N=67) and control (N=66) groups. The intervention group received a dynamic seat cushion to encourage postural shifts during sitting, while the control group received a placebo seat pad. Primary outcomes were 6-month incidence of neck and low-back pain. Secondary outcomes included sitting discomfort, pain intensity, disability, and trunk muscle performance. Analyses utilized Cox proportional hazard models.

**Results:**

During the 6-month period, 15% of participants in the intervention group developed neck pain and 10% developed low-back pain. For the control group, this was 65% and 59%, respectively. Hazard rate (HR) ratios, after adjusting for biopsychosocial factors, indicated a protective effect of the intervention for neck pain [HR_adj_ 0.19, 95% confidence interval (CI) 0.09–0.39, P<0.001] and low-back pain (HR_adj_ 0.16, 95% CI 0.07–0.35, P<0.001). The intervention group demonstrated a significant reduction in sitting discomfort and improvement in trunk muscle performance compared to the control group (P<0.05). However, the intervention did not reduce pain and disability in individuals experiencing pain compared to the control group.

**Conclusions:**

The dynamic seat cushion effectively reduced the incidence of neck and low-back pain by promoting postural shifts. These findings suggest that the key factor in reducing the risk of developing neck and low-back pain is the facilitation of postural shifts during sitting, which can potentially be achieved with other dynamic interventions designed to reduce prolonged and static sitting among office workers.

Work-related musculoskeletal disorders (MSD) are a significant global contributor to disability and a major reason for the demand for rehabilitation (1). Among 417 computer-using office workers in 15 financial organizations in China, who commonly engage in prolonged sitting, the point prevalence rates of mild-to-severe levels of pain, as measured by the Northwick Park neck pain questionnaire, are up to 86.3% for non-specific neck pain (NP), 75.5% for non-specific low-back pain (LBP), and 71.5% for both NP and LBP (2). A meta-analysis revealed a positive association between prolonged workplace sitting and the occurrence of NP and LBP (3). Furthermore, the impact of prolonged sitting at work is associated with various physical and psychological deleterious health outcomes (4, 5). Consequently, NP and LBP can lead to reduced work productivity, sickness absenteeism, increased medical and pharmaceutical expenses, and result in a significant socio-economic burden on both patients and society (6).

Prolonged sitting at work has been associated with gradual increase in discomfort across various parts of the body, which in turn may be associated with the development of NP and LBP (7, 8). Prolonged sitting is defined as sitting or reclining with an energy expenditure of ≤1.5 metabolic equivalents (MET) for ≥2 hours at a time. This definition applies across various domains, including work (eg, desk jobs, computer use, meeting), and leisure-time activities (eg, reading, socializing) (5). Office workers are recommended to take breaks from prolonged sitting and engage in regular exercises to reduce the risk of NP and LBP (8). However, the nature of their work makes it difficult for them to disengage from their workstations. Hence, due to the prolonged sitting, office workers often experience physical inactivity during work. Recognizing the challenges of prolonged sitting, dynamic sitting is frequently suggested as an intervention designed to encourage postural shifts or subtle movements during prolonged sitting (9, 10). However, dynamic chairs are expensive and might thus pose accessibility challenges for the general office worker. Using a gym ball might be a choice; it can enhance spine motion and trunk muscle activation (11) and even improve core muscle endurance (12). However, previous studies have reported an increase in discomfort, possibly due to the lack of a backrest when sitting on a gym ball (11, 13). Another option is sitting on a dynamic air-filled seat cushion, which has the potential to encourage subtle movement, increase tissue blood circulation, enhance maximum oxygen saturation values, and improve pressure distribution beneath the seated area (14, 15). The air-filled seat cushion may address some of the challenges associated with prolonged sitting. In a recent prospective RCT, interventions encouraging postural shifts during sitting proved effective in reducing the occurrence of new onset NP and LBP among office workers (16). To clarify, postural shifts during sitting are defined as spinal micro-movements that are supposed to cause significant changes in the load between the ischial tuberosities (17). Shifting 10-30 times per hour during sitting has been found to be effective in alleviating bodily perceived discomfort, which serves as a predictor for the risk of NP and LBP (18). Weight-shifts during sitting on an unstable surface enhance trunk muscle activation and postural control (19). Therefore, promoting postural shifts during sitting through the use of a dynamic seat cushion may be an effective intervention in reducing the onset of NP and LBP.

The aforementioned study investigating interventions involving postural shifts during sitting utilized an automatic air-pumping cushion to prevent NP and LBP among office workers (16). However, in the aforementioned study, participants were required to work from home due to the COVID-19 pandemic and were unable to set up the seat in their home. Furthermore, an automatic air-pumping cushion might still be rather expensive. We designed a new, portable dynamic air-filled cushion with a size, design, and air pressure, optimized through repetitive testing, to be placed on top of an office chair (20). However, positive results in the laboratory cannot be generalized to the real world. Therefore, the aim of the present study was to evaluate the effectiveness of this newly designed dynamic air-filled seat cushion on the onset of NP and LBP during a 6-month follow-up in a real office environment among healthy office workers with a high risk of NP and LBP. All participants worked exclusively at their workplace, ie, they were not teleworking from home or elsewhere, to maintain a controlled environment and ensure consistent ergonomic adjustments.

## Methods

### Participants

Seven organizations in Bangkok, including two universities, the Thai Court of Justice head office, and four private companies, participated in this study. Eligibility criteria included: (i) age 23–55 years; (ii) body mass index (BMI) of 18.5–22.9 kg/m^2^; (iii) ≥5 years of experience in the current job position; (iv) being at risk of non-specific NP as evaluated by the Neck Pain Risk Score for Office Workers (NROW; score ≥2) (21); and (v) non-specific LBP as determined by the Back Pain Risk Score for Office Workers (BROW; score ≥53) (22). Participants used standard office chairs equipped with 5 wheels, adjustable height, a fixed or adjustable backrest, and armrests.

Participants with any of the following criteria were excluded from the study: (i) reported NP or LBP within the past 6 months; (ii) exhibited signs of neurological deficits, such as muscle weakness or loss/disturbance of sensation; (iii) had hemorrhoids, wounds, or contusions in the buttocks or posterior thigh region; (iv) had a history of trauma or accidents in the spinal region; (v) had undergone spinal, intra-abdominal, or femoral surgery; (vi) were diagnosed with cancer, tumors, congenital anomalies of the spine, rheumatoid arthritis, infections of the spine or discs, ankylosing spondylitis, spondylolisthesis, spondylosis, osteoporosis, gout, osteoarthritis, systemic lupus erythematosus, or kidney disease; (vii) reported pregnancy; or (viii) engaged in regular physical exercise (ie, continuously >30 minutes per session, three times per week).

### Description of intervention

Seven organizations were cluster-randomized using computer-generated randomization. The number of employees in each organization ranged from 400 to 5000 persons. Each organization was designated to either the control or intervention group. To prevent contamination, each organization received only one assignment. The control group comprised one public university (N=20), the Court of Justice head office (N=21), and two private companies (N=17 and N=8). The intervention group included one private university (N=36) and two private companies (N=25 and N=6). Participants within each organization were not informed of their group assignment (control or intervention) to minimize bias.

After obtaining official organizational approval, we collaborated with human resources (HR) departments to distribute an invitation email to their employees. The email and internal channels – such as the intranet, posters, and staff meeting presentations – detailed the study’s objectives, inclusion and exclusion criteria for participation, data collection procedures, benefits of the study, participant rights, disclosure of information, and compensation for participation. Participation was voluntary, and interested workers accessed a secure online platform to complete a screening questionnaire. Responses to the screening questionnaire were screened for eligibility, and eligible participants were contacted via telephone to schedule an in-person meeting where they received comprehensive information about the study, signed informed consent forms, and completed baseline assessments.

At baseline, participants’ hip breadth was measured to determine the appropriate size for the seat cushion or placebo seat pad (within the range of 36–42 cm). The cushion size was selected based on each individual’s hip breadth measured during sitting in both groups.

Participants in the control group received a placebo seat pad made of polypropylene foam, placed on top of the chair seat, whereas the intervention group received a newly designed dynamic air-filled seat cushion, which provides an unstable seating surface aimed at encouraging postural shifts. A practical sitting test was conducted for two weeks before data collection to reduce potential discomfort associated with adopting a new, dynamic sitting position (12, 23).

When adding a seat cushion or pad, the researcher (SC) adjusted the computer workstation and chair following standard guidelines and made additional minor adjustments for each participant’s comfort. Although the dynamic seat cushion and seat pad did not look the same, participants did not know whether they were randomized into the control or intervention group. Both groups were asked to follow the provided instructions (supplementary material, www.sjweh.fi/article/4184, Appendix 1) until completing the 6-month follow-up.

### Outcomes measurement

At baseline, participants completed a self-administered questionnaire covering biopsychosocial characteristics, including individual, work-related physical, and psychosocial factors (supplementary material, Appendix 2).

Participants were asked to record a self-administered diary every week (supplementary material, Appendix 3). They documented the number of hours they used the cushion per workday, and their neck and lower-back discomfort using the Borg CR-10 scale. At the end of each month, participants were asked to record any new onset of NP or LBP that occurred over the month. If such pain occurred, they were instructed to note its intensity (Visual Analog Scale; 0–10) and disability. Participants reported NP using the Neck Disability Index (NDI; 10 items) (24), and LBP using the Roland-Morris Low Back Disability Questionnaire (RMDQ; 24 items) (25).

Participants underwent standardized physical examinations at baseline and 6 months, including the Biering-Sorensen test for lumbar erector spinae and multifidus endurance (26), the plank endurance test for assessing core muscle endurance in a functional manner (27), and the lumbar stability test to measure lumbar stability level (28). The inter- and intra-rater reliability of physical examiners, blinded to the group assignments, were assessed before collecting the data, and the results indicated good to excellent reliability (supplementary material, Appendix 4).

### Statistical analysis

All statistical analyses were performed using SPSS Statistics software, version 29.0 (IBM Corp, Armonk, NY, USA). Baseline characteristics of participants in the intervention and control groups were compared using independent t-tests for continuous data and chi-square (Χ^2^) tests for nominal and ordinal data. The analysis followed an intention-to-treat approach, and missing data were handled using hot-deck imputation. The 6-month incidence rate of NP and LBP was calculated for each group as the proportion of new-onset cases. The follow-up data for individuals initially identified as cases were not utilized further in the analysis.

Kaplan–Meier survival curves were used to measure the survival probability and compare the intervention and control groups using the log-rank test. Survival time was defined as the time (in months) to the onset of symptoms, with participants who left the study without symptoms no longer recorded.

The Cox proportional hazards model was used to calculate hazard ratios (HR) regarding incident cases of NP and LBP. Age, gender, and psychological scores were included as covariates in all models to mitigate confounding from these factors. The remaining 41 possible covariates were individually assessed in multivariate models. If a tested covariate resulted in a change of ≥0.05 in the HR of the intervention variable, it was included in the final adjusted model.

A two-way analysis of variance (ANOVA) was used to investigate the effects of group, time, and their interaction on discomfort and trunk muscle performance. Bonferroni post hoc analysis was conducted for pairwise comparisons. The independent t-test was employed for normally distributed data, while Mann–Whitney tests were used for non-normally distributed data to assess differences in duration to using the seat cushion, and pain intensity and disability among participants reporting NP or LBP in these two groups.

## Results

**Figure 1:**
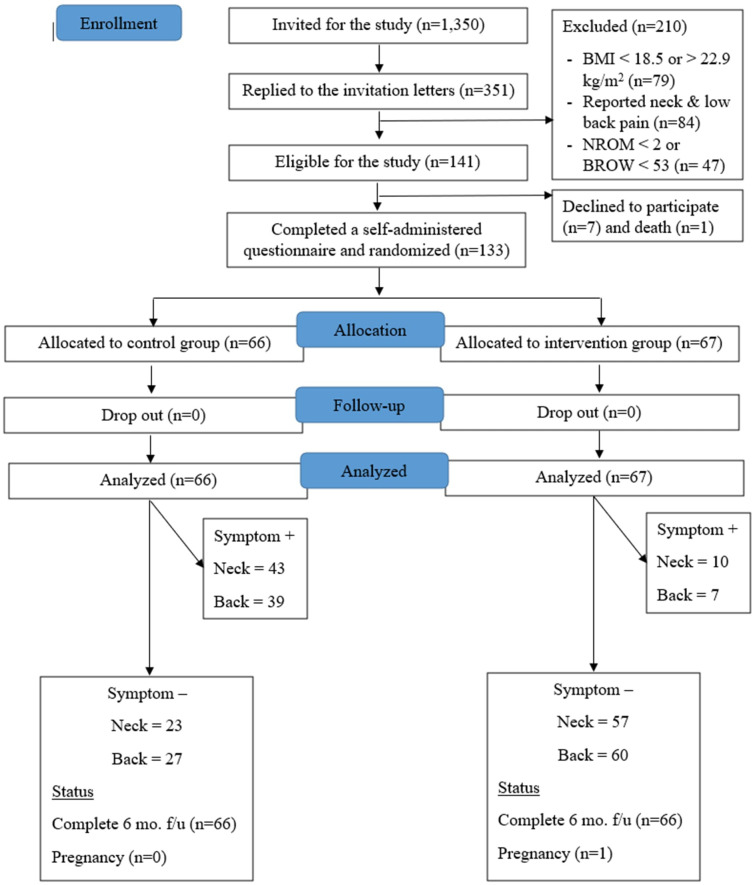
Flowchart of the study.

The trial started in April 2023 and completed in December 2023. Figure 1 illustrates the participant flow procedure in this study as proposed by the Consolidated Standards of Reporting Trials (CONSORT). In total, 1350 office workers received invitations, resulting in a 26% response rate (N=351), and 133 participants met the criteria and were included in the study. Of these, 5 participants changed jobs but remained office-based. When they started new jobs, the researcher (SC) followed them up to adjust their workstations to fit with the seat cushion or pad for each participant in the new company. A participant became pregnant at the 4^th^ month of data collection, and an intention-to-treat approach was used in the analysis. No serious harm was reported in relation to the intervention or the procedure. The baseline characteristics of the participants in the intervention and control groups are outlined in table 1.

**Table 1:** Characteristics of participants (N=133). [SD=standard deviation].

Baseline characteristic		Dynamic seat cushion (N=67)		Control (N=66)	P-value
		Mean (SD)		Mean (SD)	
Demographic					
	Age (years)	41.0 (8.0)		37.5 (7.7)	0.48
	Gender: female (%)	80.5		89.6	<0.001 *
	Body mass index (kg/m^2^)	21.2 (1.5)		20.8 (1.6)	0.20
	Hip breadth (cm)	38.3 (2.5)		37.5 (2.5)	0.64
	Education (%)				0.27
	<Bachelor’s degree	0		6.0	
	Bachelor’s degree	71.2		73.1	
	>Bachelor’s degree	27.8		20.9	
Exercise frequency in the past 12 months (%)					
	Never	55.2		43.9	
	Occasionally	44.8		56.1	
Work-related					
	Employment (years)	15.4 (8.2)		10.0 (7.3)	0.04 *
	Working hours per day	8.3 (1.0)		8.1 (1.3)	0.87
	Rest time per day (min)	58.2 (13.7)		59.5 (16.2)	0.52
Psychosocial					
	Job control	35.8 (4.5)		37.6 (5.6)	0.01 *
	Psychological job demands	32.3 (4.4)		33.2 (5.0)	0.08
	Physical job demands	13.1 (2.3)		14.1 (3.3)	<0.001 *
	Job security	9.9 (1.0)		10.5 (1.2)	0.08
	Social support	37.5 (4.5)		38.0 (5.7)	0.01 *
	Hazards at work	14.8 (2.4)		15.8 (4.0)	<0.001 *
Spinal discomfort					
	Neck	1.88 (0.98)		1.83 (1.01)	0.87
	Lower back	1.57 (0.90)		1.48 (0.86)	0.11
Trunk muscle performance assessment					
	Plank endurance test (sec)	22.43 (12.76)		35.70 (25.85)	<0.001 *
	Biering-Sorensen test (sec)	23.59 (12.97)		34.04 (20.80)	<0.001 *
	Lumbar stability test	1.66 (0.88)		1.79 (0.78)	0.06

*P<0.05

### Incidence of NP and LBP

Over the 6-month follow-up period, participants in the control group exhibited a higher incidence of NP (65%) and LBP (59%) compared to those in the intervention group (15% and 10%, respectively) (table 2). Using a Cox proportional hazard model, after adjusting for variables, protective effects of dynamic seat cushion were found for NP and LBP. The dynamic seat cushion significantly reduced the risk of incident NP (HR_adj_ 0.19) and LBP (HR_adj_ 0.16) (table 2). Thus, sitting on a dynamic seat cushion can reduce the risk of developing NP by 81% and the risk of developing LBP by 84%.

**Table 2:** Unadjusted and adjusted hazard ratios (HR) evaluating the effects of dynamic seat cushion on incident neck pain and low-back pain (N=133), along with pain and disability score of participants reporting pain during 6-month follow-up. [CI=confidence interval; SD=standard deviation].The control group is the reference group.

		Incidence		Unadjusted	P-value	Adjusted ^a^	P-value	Pain intensity		Disability
		N (%)		HR (95% CI)		HR (95% CI)		Mean (SD)		Mean (SD)
Neck pain										
	Control (N=66)	43 (65.15)		1.00		1.00		3.68 (0.93)		6.83 (2.66) ^b^
	Intervention (N=67)	10 (14.93)		0.20 (0.10–0.39)	<0.001 *	0.19 (0.09–0.39)	<0.001 *	3.68 (0.93)		6.83 (2.66) ^b^
Low-back pain										
	Control (N=66)	39 (59.09)		1.00		1.00		3.86 (1.23)		3.29 (1.71) ^c^
	Intervention (N=67)	7 (10.45)		0.15 (0.07–0.33)	<0.001 *	0.16 (0.07–0.35)	<0.001 *	3.62 (0.69)		2.67 (3.03) ^c^

*P<0.05. ^a^ Variables; age, gender, psychological demands. ^b^ Neck disability index score. ^c^ Roland-Morris low back disability score.

**Figure 2:**
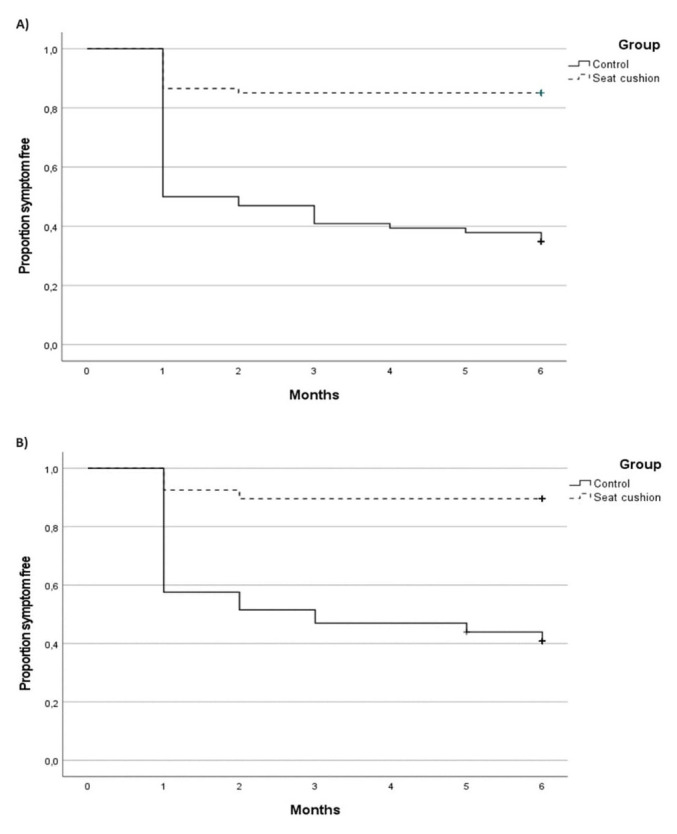
The Kaplan–Meier survival curves for A) onset of neck pain; B) onset of low-back pain.

The Kaplan–Meier survival curves for the neck and low-back cohort revealed a significant difference in the time to NP and LBP between groups (log-rank test probability <0.001) (figure 2). Participants in the control group exhibited a higher risk of experiencing NP and LBP compared to those in the intervention group.

The intervention group used the dynamic seat cushion on average 4.36 [standard deviation (SD) 1.61] hours per day, significantly lower than the control group used the placebo seat pad average use was 4.62 (SD 2.40) hours per day (P<0.001). Additionally, there were no statistically significant differences in pain intensity and disability in participants who reported NP and LBP between groups (P>0.05) (table 2).

### Effectiveness of dynamic seat cushion on discomfort and trunk muscle performance

Two-way ANOVA indicated a significant interaction effect between group and time on neck and the lower-back discomfort (P<0.001). The analysis of groups at each time point indicated that neck and lower-back discomfort in the intervention group was significantly lower than in the control group from the 1^st^ to 6^th^ month (P<0.001). The post hoc Bonferroni test indicated that neck discomfort scores in the control group showed no significant difference compared to baseline, while in the intervention group, they were significantly reduced at the 1^st^ to 6^th^ month follow-up. For the lower back, discomfort scores significantly increased during the 1^st^ month in the control group (P<0.001) compared to baseline, whereas lower-back discomfort scores in the intervention group significantly reduced at the 1^st^ to 6^th^ month follow-up (table 3, supplementary material, figure S1).

**Table 3:** Discomfort and trunk muscle performance at baseline and 6-month follow-up for the dynamic seat cushion (N=67) and control group (N=66). [FU=follow-up.]

		Interaction effect (F); P-value	Within-group changes (Baseline versus at 6-month FU)			Between-group changes at 6-month FU (Dynamic seat cushion versus control)	
			Mean difference	P-value		Mean difference	P-value
Neck discomfort		F(4.12,267.61) = 19.25, P<0.001				1.21	<0.001 *
	Dynamic seat cushion		1.28	<0.001 *			
	Control		0.04	1.00			
Lower back discomfort		F(4.08,264.90) = 25.08, P<0.001				1.25	<0.001 *
	Dynamic seat cushion		1.00	<0.001 *			
	Control		-0.45	<0.001 *			
Plank endurance test (sec)		F(1,65) = 86.05; P<0.00 *				-7.12	0.04 *
	Dynamic seat cushion		-14.19	<0.001 *			
	Control		6.18	<0.001 *			
Biering-Sorensen test (sec)		F(1,65) = 91.91; P<0.00 *				-17.39	<0.001 *
	Dynamic seat cushion		-17.39	<0.001 *			
	Control		9.40	<0.001 *			
Lumbar stability test		F(1,65) = 16.98; P<0.00 *				-0.50	<0.001 *
	Dynamic seat cushion		-0.59	<0.001 *			
	Control		0.03	0.69			

* P-value <0.05.

Two-way ANOVA showed a significant interaction effect between group and time on three physical examinations (table 3). In within-group comparisons with post hoc analysis, a significant increase in three physical examinations was observed at the 6-month follow-up in the intervention group (P<0.001), whereas only plank endurance and Biering-Sorensen were significantly reduced at the 6-month follow-up in the control group (P<0.001). Between-group comparisons with post hoc analysis showed that the intervention group exhibited a significantly greater improvement in three physical examinations at the 6-month follow-up compared to the control group (P<0.05).

## Discussion

This study aimed to evaluate the effectiveness of the promotion of postural shifts using a dynamic seat cushion on the 6-month incidence of NP and LBP among high-risk office workers. During the 6-month follow-up, participants in the control group showed an incidence of NP at 65% and LBP at 59%, whereas participants in the intervention group developed NP at 15% and LBP at 10%. Our findings suggest that using a dynamic seat cushion for a 6-month period significantly reduced the risk of developing NP by 81% and LBP by 84% among high-risk office workers. A dynamic seat cushion also decreased neck and lower-back discomfort as well as enhanced trunk muscle performance. However, sitting on a dynamic seat cushion did not show any significant benefit in reducing pain intensity and disability levels.

### Incidence of NP and LBP

During the 6-month follow-up, participants in the control group exhibited a high incidence of NP and LBP, 65% and 59%, respectively, which was higher than rates (33–44%) observed in previous studies (16, 29). The discrepancy between the present and previous studies may be attributed to differences in the baseline characteristics. In our study, the control group had an average age of 38 years, older than participants in earlier studies where the average age ranged from 30–34 years. Incident cases were higher in the age group of 35–39 compared to 30–34 years (30). Additionally, in our study, almost half (44%) reported no physical exercise in the past 12 months, whereas only 22–24% reported this in the previous studies. Physical inactivity could be a risk factor contributing to increased chances of NP and LBP (31). Consequently, the observed high incidence of NP and LBP in our study may be partly attributed to factors such as a higher average age and the influence of physical inactivity.

Our study showed that participants using a dynamic seat cushion experienced an 81–84% reduction in onset of NP and LBP compared to those in the control group during the 6-month follow-up. These results align with a prior study conducted by Waongenngarm et al (16), which demonstrated that using an automatic air-pumping seat cushion to encourage postural shifts can reduce the incidence of NP and LBP by 59–81% among high-risk office workers over a 6-month period. Participants included in both studies were office workers at high risk of NP and LBP without pre-existing symptoms. This approach is aimed at improving the allocation of interventions, directing them toward those in greater need and who are more likely to benefit (32). Additionally, the cushion’s design, similar to a gym ball, creates an unstable surface to encourage postural shifts. Each postural shift during sitting can increase subcutaneous oxygen saturation by an average of 2.2%, indicating a positive effect on tissue viability (17). Previous systematic reviews reported that dynamic sitting approaches are not effective as stand-alone management approaches for LBP (33). However, our study differs from other relevant studies in various aspects. In our study, we selected a dynamic seat cushion tailored to individual hip breadth, ensuring both suitability and comfort during sitting. Tailoring the chair design to the individual needs may be more effective than using a one-size-fits-all approach (34), which might also hold true for a seat cushion. Personalized, custom-made seat cushions offer users who spend prolonged periods in sitting adjustments that bridge the gap between ergonomic principles and practical application (35). Workstation incompatibility may increase the risk of musculoskeletal disorders among computer users (36). Thus, custom-made seat cushions may enhance outcomes by reducing discomfort, potentially lowering the incidence of NP and LBP. Furthermore, one of these articles (13) found that sitting on a stability ball with no backrest increased lower-back discomfort over time, possibly due to the lack of backrest support during prolonged period. Sitting on a chair with a backrest reduces paraspinal muscle activation and decreases lower-back discomfort (34). In our study, we employed standard office chairs with backrests and adjusted workstations according to ergonomic guidelines, with additional minor adjustments for participant comfort. This approach ensured consistent and ergonomic seating arrangements, minimizing variations from other factors and facilitating accurate assessment of the effects of dynamic seat cushion and placebo seat pad on outcomes. The combination of an air-filled cushion used with a backrest could be a key factor influencing the effectiveness of the postural shift intervention (37). Therefore, sitting on this dynamic seat cushion to encourage postural shift that is tailor-made to match the individuals’ hip breadth and using it on a chair with a backrest can reduce the risk of NP and LBP.

Participants using a dynamic seat cushion reported an average sitting time of 4.36 hours per day. Despite both groups having similar average sitting times of approximately 8 hours per day in the workplace, compliance with using the cushions revealed that participants in both groups used them for an average of 4–5 hours per day, suggesting a moderate level of adherence to the intervention protocol. The findings are consistent with a previous study that utilized a dynamic smart seat cushion and reported an average sitting time of 4.38 hours per day and a frequency of postural shifts during sitting of 27.3 times per hour (16). However, our study did not measure the frequency of postural shifts during sitting. Instead, we relied on comments from participants in the intervention group who reported that sitting on the dynamic seat cushion helped encourage postural shift. Our review revealed limited scientific evidence supporting a specific duration of using dynamic sitting for prolonged periods during computer work. Previous studies in laboratory settings have reported that sitting on a gym ball/air-filled cushion for 1–2 hours increases spinal motion, and back extensor activation (11), promotes a more neutral lumbar posture (13), and reduces discomfort (23). Sitting on a gym ball, instead of a chair, for 90 minutes per day for 8 weeks has been shown to improve core muscles endurance (12). Despite our study reporting a higher average sitting time on the dynamic seat cushion compared to previous studies, we lack information on the optimal duration for each sitting session. Therefore, future research should consider adding outcomes to assess sitting behavior (eg, sitting trackers) while using a dynamic seat cushion to better understand how the intervention may be habitually connected to other behaviors and contextual cues and identify the most effective time to implement the intervention.

Those participants who reported NP and LBP in the intervention group showed no significant difference in pain intensity or disability levels compared to the control group. In this study, cases were defined based on participants reporting NP or LBP lasting >24 hours within the past month, accompanied by a pain intensity rating of >3 out of 10 on the Visual Analog Scale, and no associated weakness or numbness in the upper or lower limbs. Monthly assessments were chosen deliberately to offer a comprehensive overview of pain occurrence over time, while also minimizing participant burden. This approach aimed to capture variations and patterns in pain experience that might not be discernible with less frequent assessments. Our result is consistent with a previous study that involved sitting on a gym ball instead of using a chair (12). The aim of the dynamic seat cushion in our study was to prevent the occurrence of NP and LBP. However, it might not be effective for treatment interventions that focus on alleviating existing pain intensity and disability problems. Future research should examine the impact of dynamic seat cushion intervention in those with NP and LBP.

### Effectiveness of a dynamic seat cushion on discomfort and trunk muscle performance

Our results showed that neck and lower-back discomfort scores significantly decreased in the intervention group, whereas neck and lower-back discomfort scores significantly increased in the control group starting from the first months. These findings align with the observations of one-third of the participants (33%) in the control group, who commented that the placebo seat pad appeared identical to the chair’s seat pan, with no noticeable difference reported. On the other hand, participants in the intervention group experienced initial instability during the first day of use. Over the 2-week training phase, they gradually increased their time using the dynamic seat cushion, following the protocol. As a result, more than half of the participants (55%) in the intervention group reported comfort and learned how to move in a way that suited them individually while sitting on it. One possible explanation for reduced discomfort while sitting on a dynamic seat cushion is that postural shifts may have minimized discomfort during prolonged sitting. Prolonged sitting in a constrained or fixed posture leads to an increase in perceived musculoskeletal discomfort over time (38, 39). Sitting for longer than 30–80 minutes may increase the risk of neck and low back pain (7). Therefore, using a dynamic seat cushion would promote postural shifts before discomfort becomes noticeable, potentially reducing neck and lower-back discomfort. These alternating movements or postural shifts, which occur during sitting on unstable seating, served as a mechanism to release pressure beneath the siting area (14) and facilitate the flow of fluids and nutrients, thereby enhancing tissue viability (17). This could be a contributing factor to discomfort reduction. Another possible factor is that sitting on an air-filled seat cushion may relieve interface pressure on compressed areas, similar to manual offloading approaches (14) such as manual postural adjustment or using cushions to redistribute weight, leading to improved blood perfusion and reduced discomfort. Thus, our results suggest that utilizing an air-filled seat cushion, which promotes postural shifts to break up static prolonged sitting, may contribute to the reduction of neck and lower-back discomfort.

Our results demonstrated that sitting on a dynamic seat cushion can improve core trunk muscle endurance and lumbar stability, while the control group showed a significant decrease in trunk endurance at the end of the 6-month follow-up period. These findings are consistent with a previous study that showed sitting on a gym ball for 8 weeks improves core muscles endurance (12). An explanation for the observed enhancements in core muscle endurance and lumbar stability could be attributed to the influence of the air-filled unstable surface of the dynamic seat cushion. This feature promotes postural shifts and movements during sitting, potentially enhancing trunk muscle activation. Previous studies have shown that sitting on gym ball can effectively engage various core muscles, demonstrating minimal muscle activity ranging from 0.51–5.56% maximum voluntary isometric contraction (11, 13, 40), while also potentially leading to muscle fatigue due to insufficient changes in muscle tissue oxygenation (41). It is important to note that the dynamic seat cushion utilized in our study actively promotes movements during sitting. These movements are designed to induce changes in muscle fiber recruitment patterns, potentially preventing prolonged static muscular contraction that contributes to muscle fatigue. Improving muscle endurance through training often involves exercises against a relatively low load performed over a long-term period (42). This concept is particularly relevant to our study, considering the dynamic seat cushion’s role as a tool for facilitating muscle endurance improvement through postural shifts, which might be considered low-load exercise and training over 6 months. The alternating movement may facilitate training core muscles’ co-contraction to improve spinal stability (43). Therefore, the dynamic seat cushion, serving as a light-exercise device, facilitates shifting movements during sitting in multiple directions.

### Observations and practical insights

Remarkably, the intervention group displayed positive outcomes, in contrast to the potentially worsening outcomes in the control group. This disparity may be influenced by significant baseline differences in psychosocial aspects of work and core muscle endurance between the two groups, particularly with participants from the Court of Justice head office in the control group, potentially experiencing higher levels of work-related stress. Psychological and organizational factors at work have been linked to disability retirement rates (44). These baseline disparities could obscure the true effect of the intervention, potentially exaggerating its perceived effectiveness. Additionally, the phenomenon of regression to the mean, where extreme baseline scores tend to normalize over time, might partly explain the observed worsening outcomes in the control group. Future studies should consider matching organizations based on their baseline characteristics to ensure comparable groups at the outset of the study (45). This approach is crucial for controlling baseline variability and obtaining a clearer understanding of the intervention’s true impact. Utilizing wait-list control clinical trials as a control intervention could be a prudent choice, given the potential for wait-list control clinical trials to significantly influence the perceived effectiveness of the treatment (46).

Furthermore, in settings where the seat cushions used in our study are unavailable, adopting the principles of using a custom-made seat device that encourages postural shifts while using an office chair with a backrest can significantly alleviate discomfort. Our findings indicate that after 6 months of use, this approach not only serves as a light-exercise tool but also shows promise in preventing NP and LBP.

### Strengths and limitations

A major strength of this study is its prospective cluster-RCT design, evaluating interventions to prevent the occurrence of NP and LBP, and that the intervention was assessed within an office workplace, resembling real-world conditions. Both groups had their computer workstations adjusted according to standard guidelines, and a placebo seat pad was utilized in the control group. Furthermore, our study employed double-blinding (participant and physical examiner). These workstation adjustments, utilization of a placebo seat, and double-blinding may have mitigated biases and the placebo effect’s influence on the study outcomes, thereby enhancing the internal validity of the research findings. However, our investigation has several limitations that should be taken into consideration when interpreting the results of our study. Firstly, we assessed work-related psychological factors, sitting time using the cushion, discomfort, pain, and disability using a self-report scale, introducing potential bias (47). However, we employed additional objective measures, such as physical examinations, to enhance the validity of these results beyond self-report data. Secondly, our study focused on high-risk, healthy office workers in the Bangkok area, Thailand, while controlling for specific workplace characteristics. Therefore, caution is necessary when applying our findings to other regions or populations. The effectiveness of seat cushions in reducing NP and LBP may vary depending on these contextual factors. Future research in diverse settings is recommended to confirm the broader applicability of our results. Thirdly, the relatively small sample size (N=133) may increase the likelihood of a type II error, and the study’s restriction to organizations in Bangkok area may limit generalizability, despite having an 80% statistical power to detect moderate to large effects. Finally, this study exclusively involved participants working at their workplace and controlled for the characteristics of standard office chair. This approach was chosen to manage environmental factors, minimize potential confounding variables that could affect study outcomes, and maintain consistency in the workplace conditions where the cushions were assessed. It is important to note that caution should be exercised when extrapolating these findings to teleworking, work-from-home setups, or other types of chairs. Further research is recommended to investigate the effects of dynamic seat cushions on populations experiencing NP and LBP. This includes exploring its impact across diverse occupational settings, including teleworking environments, and different regions. Such studies aim to enhance our comprehensive understanding of the potential benefits of dynamic seat cushions to prevent NP and LBP.

### Concluding remarks

Our results indicate that sitting on a dynamic seat cushion during work can effectively reduce the risk of developing NP by 81% and LBP by 84%, alleviate discomfort, and enhance trunk muscle performance. However, the dynamic seat cushion did not reduce pain intensity and disability in individuals experiencing NP and LBP. This study suggests that the observed benefits, including reduced incidence of NP and LBP, discomfort, and improved trunk muscle performance, are likely attributable to the promotion of postural shifts facilitated by the dynamic seat cushion. These postural shifts counteract the negative effects of static prolonged sitting and can help prevent NP and LBP among high-risk office workers. Therefore, it is plausible that similar results could be achieved with other dynamic interventions that also promote regular movement, thus reducing prolonged and static sitting.

## Supplementary material

Supplementary material
