# Supplementary material for: The effectiveness of a dynamic seat cushion in preventing neck and low-back pain among high-risk office workers: a 6-month cluster-randomized controlled trial
Source: Scand J Work Environ Health. 2024 Sep 30;50(7):555–66. doi: 10.5271/sjweh.4184 (PMC11479679; doi:10.5271/sjweh.4184)
Supplement: Supplementary material [file SJWEH-50-555-S001.pdf]

The effectiveness of a dynamic seat cushion in preventing neck and low back pain among high-risk office workers: a 6-month cluster-randomized controlled trial[1]

by Sirinant Channak, MSc, Erwin M. Speklé, PhD, Allard J. van der Beek, PhD, Prawit Janwantanakul, PhD[2]

1. Supplementary material
2. Correspondence to: Prawit Janwantanakul, Department of Physical Therapy, Faculty of Allied Health Sciences, Chulalongkorn University, Bangkok, 10330, Thailand [Email: prawit.j@chula.ac.th].

Appendix 1: Instructions for Participants

The researcher (SC) adjusted the computer workstation and chair according to standard ergonomic guidelines (1) and made additional minor adjustments for each participant's comfort. The seat height was set so that hips and knees were flexed at 90 degrees, with feet flat on the floor or supported by a footrest. The monitor was positioned so that the eye level was at the top of the main monitor or 1-2 inches below. Elbows were flexed at 90 degrees and supported by armrests, while wrists were kept flat on the keyboard and resting on the desk. Important items were placed in the most accessible positions, with the main computer monitor directly in front of the participant and at arm's length away.

<u>Control Group</u>

During the 6-month follow-up period:

- Participants were instructed to sit as they normally would.
- Participants were instructed to use the seat pad throughout the entire workday for the duration of the 6-month follow-up and received a notification message at the beginning of each workday to remind them to use the seat pad.
- Participants were instructed to maintain their usual level of physical activity, avoid additional physical exercise, and follow the provided instructions until completing the 6-month follow-up.

<u>Intervention Group</u>

First Two Weeks (Practical Sitting Test):

- Participants were instructed to use the cushion for 30 minutes in the morning and 30 minutes in the afternoon during work. They were gradually instructed to increase their daily sitting time on the cushion by 10% over two weeks to minimize the risk of injury associated with adopting a new, sitting position.

During the 6-month follow-up period:

- Participants were instructed to sit on the chair with the cushion and shift their body in various directions, including movement in the anterior-posterior, lateral-medial, and rotational planes on a cushion, or simply maintain balance.
- Participants were instructed to use the seat cushion throughout the entire workday for the duration of the 6-month follow-up and received a notification

message at the beginning of each workday to remind them to use the seat cushion.
- Participants were instructed to maintain their usual level of physical activity, avoid additional physical exercise, and follow the provided instructions until completing the 6-month follow-up.

Appendix 2: Self-administered questionnaire

At baseline, all participants complete a self-administered questionnaire. The self-administered questionnaire included the collection of the following biopsychosocial characteristics: individual, work-related physical and psychosocial factors.

- Individual factors included age, gender, educational level, underlying health conditions and medication use, frequency of physical exercise or sport, alcohol and tobacco habits, and number of driving hours per day.
- Work-related physical factors included working experience, working position, number of working hours, the duration of using a computer and rest breaks, performance of various activities during work, self-rating (yes or no) of the ergonomics of their workstations (i.e. the appropriateness of position of desk, chair, and monitor) and work environment conditions (light intensity, ambient noise, temperature, and air circulation).
- Work-related psychosocial factors were measured by using the Job Content Questionnaire (JCQ Thai version-54 items; rate 1: strongly disagree, to rate 4: strongly agree); psychological demands (12 items), decision latitude (11 items), social support (8 items), physical demands (6 items), job security (5 items), and hazards at work (12 items) (2).

Appendix 3: Self-administered diary

Every week, participants were asked to record a self-administered diary via a personal message at 5 PM on Friday. Participants were asked to record:

- number of hours they used the seat cushion or seat pad per workday
- overall score for neck and lower back discomfort for the week using the Borg CR-10 scale (0-10; where 0 indicates no discomfort and 10 indicates extreme discomfort) (3).

At the end of each month during the 6-month follow-up period, participants were asked to record:

- number of hours they used the seat cushion or seat pad per workday
- overall score for neck and lower back discomfort for the week using the Borg CR-10 scale (0-10; where 0 indicates no discomfort and 10 indicates extreme discomfort)
- new onset of neck or low back pain that occurred over the month.
    - If such pain occurred, they were instructed to note its intensity and any resulting disability.
        - The question about a new onset of non-specific neck or low back pain was, "Have you experienced any neck or low back pain lasting more than 24 hours during the past month?" If they answered "Yes",

then follow-up questions about pain intensity, which was measured by a visual analogue scale (VAS; 0-10), and the presence of weakness or numbness in the upper or lower limbs were administered. Those who answered "Yes", reported a pain intensity more than 3/10 VAS, and had no weakness or numbness were identified as cases.

- Participants who reported neck pain were also asked about their disability level, measured using the Neck Disability Index (NDI-10 items; total score ranges from 0 to 50, with higher scores indicating more severe neck disability) (4).
- Participants who reported low back pain were assessed using the Roland-Morris Low Back Disability Questionnaire (RMDQ-24 items; total score ranges from 0 to 24, with higher scores indicating more severe low back disability) (5).

Cases were identified if participants reported neck or low back pain lasting over 24 hours in the past month, with a pain intensity greater than 3/10 on VAS and no weakness or numbness.

Appendix 4: Physical examinations

Three physical examinations included the Biering-Sorensen test, the plank endurance test, and the lumbar stability level test. Physical examiners completed 20 hours of practical training for the examinations, and the inter-rater and intra-rater reliability were assessed for 10 workers on two separate occasions on different days.

1. The Biering-Sorensen test, a valid measure of lumbar erector spinae and multifidus endurance (6). The reliability of the Biering-Sorensen test was excellent for intra-rater reliability ($ICC_{(3,1)} = 0.92$) and good for inter-rater reliability ($ICC_{(2,1)} = 0.79$).
2. The plank endurance test is a valid, reliable and practical method for assessing core muscle endurance in a functional manner (7). The reliability of the plank endurance test was excellent for intra-rater reliability ($ICC_{(3,1)} = 0.99$) and inter-rater reliability ($ICC_{(2,1)} = 0.96$).
3. The lumbar stability level test was conducted using pressure biofeedback, following the guidelines by Thongjunjua (8). The reliability of the lumbar stability test was good for intra-rater reliability ($ICC_{(3,1)} = 0.87$) and excellent for inter-rater reliability ($ICC_{(2,1)} = 0.90$).

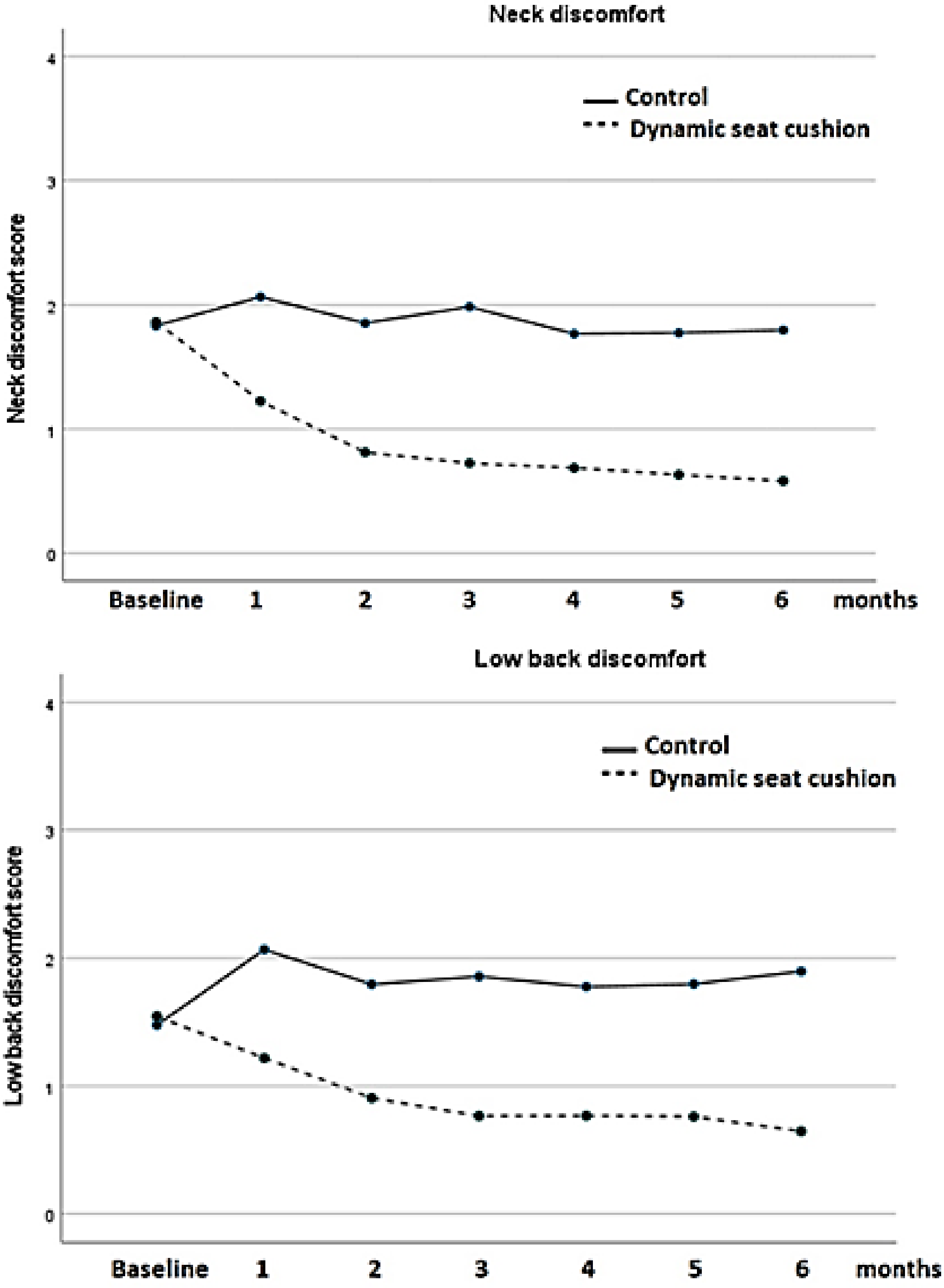

Figure S1. Mean of neck and lower back discomfort scores over the 6-month follow-up period for dynamic seat cushion (n=67), and control groups (n=66).

References

1. Sanders MJ. Ergonomics and the management of musculoskeletal disorders. Place of publication not identified: Butterworth Heinemann; 2004.
2. Bhuanantanondh P, Buchholz B, Arphorn S, Kongtip P, Woskie S. The Prevalence of and Risk Factors Associated with Musculoskeletal Disorders in Thai Oil Palm Harvesting Workers: A Cross-Sectional Study. Int J Environ Res Public Health. 2021;18(10).
3. Borg G. Psychophysical scaling with applications in physical work and the perception of exertion. Scand J Work Environ Health. 1990;16 Suppl 1:55-8.
4. Uthaikhup S, Paungmali A, Pirunsan U. Validation of Thai versions of the Neck Disability Index and Neck Pain and Disability Scale in patients with neck pain. Spine (Phila Pa 1976). 2011;36(21):E1415-21.
5. Wiangkham T, Phungwattanakul N, Thongbai N, Situy N, Polchaika T, Kongmee I, et al. Translation, cross-cultural adaptation and psychometric validation of the Thai version of the STarT Back Screening Tool in patients with non-specific low back pain. BMC Musculoskelet Disord. 2021;22(1):454.
6. Coorevits P, Danneels L, Cambier D, Ramon H, Vanderstraeten G. Assessment of the validity of the Biering-Sørensen test for measuring back muscle fatigue based on EMG median frequency characteristics of back and hip muscles. Journal of Electromyography and Kinesiology. 2008;18(6):997-1005.
7. Tong TK, Wu S, Nie J. Sport-specific endurance plank test for evaluation of global core muscle function. Phys Ther Sport. 2014;15(1):58-63.
8. Thongjunjua S, Mahidon M, Mahāwitthayālai Mahidon. Khana Phǣtthayasāt S. Effects of Lumbar Stabilization Exercises on Exercise Level Attained in Healthy Subjects2005.
